# Perceived quality of primary healthcare post-National Health Insurance pilot implementation

**DOI:** 10.4102/hsag.v26i0.1580

**Published:** 2021-05-31

**Authors:** Hillary Mukudu, Kennedy Otwombe, Caiphus Moloto, Adam Fusheini, Jude Igumbor

**Affiliations:** 1 School of Public Health, Faculty of Health Sciences, University of the Witwatersrand, Johannesburg, South Africa; 2 Perinatal HIV Research Unit, Faculty of Health Sciences, University of the Witwatersrand, Johannesburg, South Africa; 3 Health Directorate, Health Information Management, Department of Health, Johannesburg, South Africa; 4 Department of Preventive and Social Medicine, Otago Medical School, University of Otago, Dunedin, New Zealand

**Keywords:** universal health coverage, primary healthcare, outpatient department, self-referral rate

## Abstract

**Background:**

Contracting private medical practitioners for the National Health Insurance pilot project in 2012 by the National Department of Health in South Africa was envisaged to reduce workload at referral district hospitals by reducing self-referral by clients as the perceived quality of care at the primary healthcare level improves.

**Aim:**

To describe the effect of contracting private medical practitioners at primary healthcare facilities on the self-referral rate of clients at district hospitals as a proxy for perceived quality of care in a National Health Insurance pilot district.

**Setting:**

The study was set in Tshwane National Health Insurance pilot district compared to Ekurhuleni district.

**Methods:**

We compared findings before and after implementing the National Health Insurance private medical practitioners contracting between a pilot and a non-pilot district. A quasi-experimental ecological study design was used to compare district hospital outpatient department indicators of clients follow-up, self-referral, self-referral rate and referred in the two districts from June 2012 to May 2014 using single and controlled interrupted time-series analyses.

**Results:**

Controlled interrupted time series analysis found decreases in self-referral rate (−1.8 [−2.2, −1.1] [*p* < 0.0001]) and the initial trend of headcounts of self-referral (−516 [−969, −66] [*p* = 0.0260]), but an increase in headcounts of referred clients (1293 [77, 2508] [*p* = 0.0376]) in the pilot compared with the non-pilot district.

**Conclusion:**

We concluded that the implementation of contracting private medical practitioners in primary healthcare facilities might have resulted in an improved perceived quality of care at primary health care facilities. However, the higher number of outpatient department headcounts for follow-up and the increase in referred cases in the pilot district would need to be investigated.

## Introduction

Understanding the effects of the perceived quality of primary healthcare (PHC) is critical to the attainment of universal health coverage (UHC) globally (World Health Organization 2019). In 2019, the United Nations (UN) reaffirmed UHC with strong health systems based on the values of PHC for the provision of quality healthcare as a goal for member countries to attain by 2030 (United Nations 2019). Poor quality of health service has been recognised as a significant problem in developing countries (Chou 2019). In South Africa, structural challenges with the public PHC system have contributed to a perception of poor quality of services, which include a shortage of staff, poor staff attitudes, long waiting times, low standards of cleanliness, drug stock-outs, poor infection control and inadequate security for both staff and clients and the non-availability of integrated patient-level health information system for data collection and reporting (National Department of Health, South Africa [NDoH] 2015). All this is happening at the time when the country is experiencing a convergence of epidemiological challenges as a result of human immunodeficiency virus or acquired immunodeficiency syndrome (HIV or AIDS) and antiretroviral treatment, non-communicable diseases (NCDs) such as hypertension, diabetes mellitus, asthma, cancers, cardiac diseases, mental health problems and other lifestyle-related diseases and complex social and behavioural changes and injuries (NDoH South Africa 2015). The impact of these factors has been overutilisation of outpatient departments (OPD) of district hospitals as clients bypass PHC facilities to receive care at a higher level because of shortage or non-existence of medical doctors at the PHC level (Mojaki et al. 2011:2011). The National Health Insurance (NHI) pilot project implemented in Tshwane and 10 other districts in South Africa from 2012 as part of UHC, focusing on various health system strengthening interventions at the PHC level (Naidoo 2012:149–150), was envisaged to address this by contracting private medical practitioners (MPs) to provide services in PHC clinics (Moosa 2014:155–156).

There are several mechanisms through which MPs were anticipated to improve the quality of PHC services. It was envisaged that MPs would, apart from seeing PHC clients, strengthen clinical governance, reduce waiting times, do infection control inspections, drug stock checks, file reviews, nurse mentoring and training and conduct morbidity and mortality reviews (The Republic of South Africa, Department of Planning Monitoring & Evaluation 2017). The outcome of this would be to not only take-on and manage complicated cases at the PHC level beyond the capacity of nurse practitioners (NPs) but also minimise referrals to higher levels. Thus, it was hypothesised that the presence of privately contracted MPs in the PHC facilities would lead to improvement of care at the PHC level, resulting in a reduction in workload at the higher levels of care (Genesis Analytics 2019). The mechanism of this would be through MPs reducing secondary care activity by various means, including restricting referral criteria, supporting community-based care models, investing in preventative healthcare and promoting services to prevent readmissions (Department of Health 2010).

Others have hypothesised that perceived improved quality of care at PHC facilities would lead to high volumes of clients and that MPs would have an increased workload, to which they would respond by increasing referrals to the next level of care (Groenewegen & Hutten 1991:1111–1119). However, studies have shown that the increase in the utilisation of PHC services in NHI pilot districts cannot be attributed only to the contracting of private MPs (Mukudu et al. 2020). This raises questions about the appropriate measure of the quality of care in view of simultaneous implementation of other aspects of PHC re-engineering and healthcare reforms such as ward-based outreach teams (WBOTs), district clinical specialist teams (DCSTs), centralised chronic medicine dispensing and distribution (CCMDD), Ideal Clinic Realisation and Integrated School Health Programme (ISHP) (Naledi, Barron & Schneider 2011:17–28).

Definitions of quality of healthcare are varied and contested (Campbell, Roland & Buetow 2000:1611–1625), just as quality measures. However, one generally accepted definition is that it is the ‘degree to which health services for individuals and populations increase the likelihood of desired health outcomes and are consistent with current professional knowledge’ (Catumbela et al. 2013:236). By this definition, individuals’ and populations’ perceptions of quality are likely to differ, making quality measurement a contested issue because of the different approaches and models used for assessment. One essential requirement for a better quality of care is universal access. This, notwithstanding, Donabedian (2005) has stated that quality of healthcare can be measured from three components: structure, process and outcomes, which refers to the state of health or events that follow care and that may be affected by healthcare. However, the outcome of care, such as perceived quality, may not only be a component but a consequence of healthcare (Campbell et al. 2000:1611–1625). Quality of care also has an impact on the implementation fidelity of an intervention and affects the outcome (Carroll et al. 2007:1–9).

Health indicators are used as a measure of outcome by the District Health Information System (DHIS) of the NDoH. One of the indicators routinely collected at hospital OPD in the DHIS is the headcounts and rates of clients not-referred (self-referral) (NDoH 2011). Several studies in the United States of America (Liu et al. 2008:124–130) and the South African provinces of KwaZulu-Natal (Pillay & Mahomed 2019:33), Limpopo (Visser et al. 2015:333–336) and Western Cape (Becker et al. 2012:800–801), and in the district of Tshwane (Masango-Makgobela, Govender & Ndimande 2020:1–7), have shown a directly proportional relationship between self-referrals to higher levels of care and a perception of poor quality of services at lower levels of healthcare (PHC). Other related indicators at OPD are headcounts of clients referred and follow-up (NDoH 2011).

Measuring perceived quality in healthcare is important as it differentiates effective coverage from crude intervention coverage (Ng et al. 2014:9). The measurement of the perceived quality of care of a health service will help to inform the implementation of NHI in many areas, such as benchmarking and measurement of programme implementation. The perceived quality of a UHC is crucial because it bridges the gap between health system building blocks and the desired health outcomes (World Health Organization 2010). It will also help to prioritise patients’ interest and determine the perceived appropriateness of the care they receive. These data will be imperative in informing legislation and policies to govern clients’ referral from PHC to higher levels of care, suggest possible structural changes to the district health system approach and prioritise resources and capacity building. We thus set out to determine the effect of private MPs contracting as part of the NHI pilot project on the headcounts and rates of clients not referred (self-referral) at district hospitals as a proxy for perceived quality of PHC services (Visser et al. 2015:333–336). We used controlled interrupted time series analysis (CITSA) in comparing headcounts and rates of self-referral before and after implementation of the programme. We hypothesise that private MPs’ placement in PHC facilities would improve the perceived quality of care, leading to a reduction in onward self-referrals. To the best of our knowledge, this is the first study to determine the perceived quality of care at the PHC level using secondary data on effect at higher levels of care.

## Methods

### Study design

We used a quasi-experimental study design by CITSA using DHIS monthly reports (Anaby et al. 2014:457–470). This design is recommended for evaluating a population-level intervention because it considers pre-existing secular trends and that there is a clear differentiation of the pre-intervention and post-intervention periods (Bernal, Cummins & Gasparrini 2017:348–355).

### Study setting

This study was carried out in district hospitals of the Tshwane NHI pilot district, Gauteng province, South Africa, which has the largest and most diverse population in terms of socio-economic status of all the NHI pilot districts (NHI Pilot District Profiles|Health -e 2019a). The district has a population of more than 2 921 488 people receiving PHC services from 68 facilities (facility to population ratio of 1:36 980) (Health Systems Trust 2019). We compared the findings with those in a non-NHI district, Ekurhuleni, which has a higher population of 3 178 470 people with 90 PHC facilities (facility to population ratio of 1:35 316) (Metropolitan Municipality, Statistics South Africa 2019a). The selection of Ekurhuleni as a comparison district was based on the proximity of the two districts and similarities in demographic profiles (Metropolitan Municipality, Statistics South Africa 2019b). Being under the same provincial governance, a uniformity of implementation of health programmes was assumed.

### Variables and outcome measurements

We measured and compared the selected OPD indicators as they met the criteria of reflecting intended and unintended outcomes and the ability to change relatively quickly after implementing an intervention or after a clearly defined lag (Bernal et al. 2017:348–355). It was anticipated that the improvement of healthcare services in PHC facilities would offset workload in OPD clinics based at district hospitals (Nanyonjo et al. 2015:1–10). The primary outcome variable was the rate of new OPD clients not referred by a PHC facility accessing services at a district hospital OPD (self-referral rate) and the secondary outcome was the headcounts of these self-referred clients because of difference in population size between the two districts. ‘Self-referral’ in the study was defined as the sum of new clients attending a general or specialist outpatient clinic without a referral letter from a PHC facility or a doctor. The measurement was used to monitor utilisation trends of PHC clients’ bypassing clinics and community health centres (CHCs) and the effect of PHC re-engineering on OPD utilisation. The self-referral rate was calculated using self-referred clients as a proportion of the total OPD new clients. The target for both is lower numbers, as an indication of clients entering the health system at the appropriate level of care (South African NDoH 2020). Other variables collected were ‘follow-up’, defined as the sum of clients attending a general or specialist outpatient clinic for follow-up care, and ‘referred’, defined as the sum of new clients attending a general or specialist outpatient clinic with a referral letter from a PHC facility or a doctor. The complete list of variables, definitions, use, impact model and mechanism of the impact model are shown in Table 1.

**TABLE 1:** Definitions and impact model of outpatient department data elements and indicators.

Data element or indicator	Definition	Use and context	Proposed impact model	Mechanism of impact model
Self-referral: OPD headcount not referred new	New clients attending a general or specialist outpatient clinic WITHOUT a referral letter from a PHC facility or a doctor	Monitors the utilisation trends of clients who bypass PHC facilities.	Level and slope change	This is likely to reduce as patients will be seen by doctors at PHC level first as the reason for this was absence of doctors at PHC facilities.
Self-referral rate: OPD new client not referred rate	New OPD clients not referred as a proportion of OPD new clients – total. (OPD headcount not referred new) ÷ (OPD headcount not referred new) + (OPD headcount referred new)	Monitors utilisation trends of clients by-passing PHC facilities and the effect of PHC re-engineering on OPD utilisation. Do not include OPD follow-up and emergency clients in the denominator	Level and slope change	This is likely to reduce as patients will be seen by doctors at PHC level first as the reason for this was absence of doctors at PHC facilities.
Referred: OPD headcount referred new	New client attending a general or specialist outpatient clinic with a referral letter from a PHC facility or a doctor	Monitors the utilisation trends of clients who do not bypass PHC facility.	Level and slope change	This is likely to increase as doctors at PHC are required to write a referral letter when transferring patients to a higher level of care.
Follow-up: OPD headcount follow-up	Client attending a general or specialist outpatient clinic for follow-up care	Monitors utilisation of OPD services.	Level and slope change	This is likely to reduce as some patient who bypass PHC facilities because of a lack of doctors can be adequately treated and managed at PHC level by private contracted MPs.

*Source:* Department of Health, Republic of South Africa, n.d., *NIDS Integrated*, viewed n.d., from https://dd.dhmis.org/indicators.html?file=NIDS%20Integrated&source=nids

OPD, outpatient departments; PHC, primary healthcare; MP, medical practitioners

### Data quality and management

District Health Information System monthly reports from June 2012 to May 2014 for OPD headcounts of ‘follow-up’, ‘referred’, ‘self-referral’ and self-referral rate in Tshwane and Ekurhuleni districts were collated. Each district hospital collected data on the clients attending outpatient services and sent these to Health Information Management (HIM) officers who create electronic formats (Excel). The data in the DHIS were complete as the values for the elements were reported for every month for the period of the study.

### Time limits and selected data points

The unit measure for selected data points was months as per DHIS reporting. A total of 24 time periods with 12 before and 12 after implementation of MPs contracting were selected. The minimum time period required for interrupted time series analysis (ITSA) is 10 before and 10 after implementation of a programme to have at least 80% power. The selection can detect a change level of at least five standard deviations of the pre-data if the autocorrelation is > 0.4 (Kontopantelis et al. 2015:1–4). The intersection for NHI MPs contracting was informed by two annual progress reports of the NHI pilot programme for 2012 (Strengthening South Africa’s Response to HIV and Health 2013) and 2013 (Andrews et al. 2014). The 2012 report showed that despite the announcement to start the NHI pilot programme in March 2012 (NHI Pilot Districts Announced|Health-e 2019b), by the end of 2012 no private MPs had been contracted yet. However, the 2013 report showed that 55 MPs were active in the Tshwane district by the end of 2013 (Strengthening South Africa’s Response to HIV and Health 2013). We thus selected the midpoint of 2013 (June) as the intersection of the intervention.

### Statistical analysis

We compared population characteristics from 2010 to 2014 between Tshwane and Ekurhuleni districts using data from Statistics South Africa (Metropolitan Municipality, Statistics South Africa 2019b). The District Health Information System data for OPD indicators were then collated and a comparison of the means (average) of pre-and post-NHI indicators for the two districts was made. On further analytical comparison, we first did a single group ITSA of selected OPD secondary data elements from the DHIS (Department of Health 2011) to project the pre-intervention trend for the post-intervention measurement as a counterfactual for each district separately. We estimated regression coefficients by the ordinary least squares method, producing Newey–West standard errors. Regression diagnostics were tested using the Cumby–Huisinga test on the error distribution to test for residual autocorrelation, which was adjusted for by Prais–Winsten regression. This was carried out to determine whether there was a statistically significant difference between the pre-and post-intervention slopes outside the intervention period in both districts. The series of monthly counts were assumed to follow a Gaussian distribution, which is not affected by overdispersion (Bernal et al. 2017:348–355). Seasonality was accounted for using dummy variables to define each month of the year as adjustment factors in the analysis.

The formula used for this ITSA was as follows:

$$Y_{t}=\beta_{0}+\beta_{1}T_{t}+\beta_{2}T_{t}+\beta_{3}X_{t}T_{t}+e_{t}X$$ [Eqn 1]

where:

- *Y*_*t*_ is aggregate OPD headcounts measured at *t* equally spaced time points.
- *T*_*t*_ is the time elapsed since the beginning of the intervention.
- *X*_*t*_ (indicator) represents the intervention.
- *X*_*t*_*T*_*t*_ is an interaction term between OPD headcounts.
- *e*_*t*_ is the random error.
- *β*_0_ is the intercept of the line on the vertical axis (initial level of the OPD headcounts).
- *β*_1_ is the slope or trajectory of OPD headcounts before the intervention.
- *β*_2_ is the change in the level of OPD headcounts immediately after the introduction of the intervention.
- *β*_3_ is the difference between the pre- and post-intervention slopes. Therefore, significant values of *p* at *β*_2_ indicate an immediate intervention effect and significant values of *p* at *β*_3_ indicate an intervention effect over time.

We then used the Prais–Winsten Auto-Regression (AR)(1) model of regression to perform a multiple group or CITSA. This allowed us to control for both the pre-intervention trends in the outcomes and for potential confounding events that would have affected both the control and the study groups. We modelled the association as a slope change rather than as an immediate level change (sudden increase in headcounts or rates) because the selected outcomes were likely to change gradually as MPs were contracted. Multiple group ITSA was performed according to Linden’s description (Linden 2015). We estimated differences in coefficients according to the following formula:

$$\begin{array}{l} Y_{t}=\beta_{0}+\beta_{1}T_{t}+\beta_{2}X_{t}+\beta_{3}TX_{t}+\beta_{4}S+\beta_{5}ST+\beta_{6}SX_{t}+\\ \;\beta_{7}STX_{t}+e_{t} \end{array}$$ [Eqn 2]

where *S* is a dummy variable for assignment to treatment or control.

Stata statistical software package, version 16 (StataCorp, TX, USA) was used to perform all the statistical analyses.

### Ethical considerations

Ethics approval for this study was obtained from the University of Witwatersrand Human Research Ethics Committee (HREC) (Certificate number: M180956). The DHIS data are aggregated at district level, and thus it does not contain any identifiers.

## Results

Table 2 shows that from 2012 to 2014, the population growth rate and the percentage with medical insurance were slightly higher in the pilot district than in the non-pilot district.

**TABLE 2:** Population characteristics of the pilot and non-pilot districts.

Demographic characteristic	Tshwane NHI pilot district			Ekurhuleni non-pilot district		
	2012	2013	2014	2012	2013	2014
**Population** [31][24]	3 012 054	3 105 428	3 201 696	3 256 978	3 337 426	3 419 860
Population growth (%)	3.1	3.1	3.1	2.5	2.5	2.5
**Sex ratio** (males per 100 females)	99	99	99	105	105	105
**Age**						
Young (1–14 years)	23.2	23.2	23.2	24.3	24.3	24.3
Working age (15–64 years)	71.9	71.9	71.9	71.7	71.7	71.7
Elderly (≥ 65 years)	4.9	4.9	4.9	4.0	4.0	4.0
**Percentage with medical insurance** [23]	33.2	33.2	33.2	25.5	25.5	25.5
Unemployment rate	24.8	24.8	24.8	28.8	28.8	28.8
**Dependency ratio per 100** (15–64)	39.0	39.0	39.0	39.4	39.4	39.4

*Source:* Metropolitan Municipality, Statistics South Africa, 2019a, *Demographic profiles*, viewed 06 October 2017, from https://municipalities.co.za/overview/4/city-of-ekurhuleni-metropolitan-municipality; and, Metropolitan Municipality, Statistics South Africa, 2019b, *Demographic profiles*, viewed 03 October 2019, from https://municipalities.co.za/overview/3/city-of-tshwane-metropolitan-municipality

NHI, National Health Insurance

A pre- to post-NHI comparison of all measured OPD indicators (Table 3) showed increases that were more pronounced in Ekurhuleni district than in Tshwane district, except for the OPD headcounts not referred, which was reduced. Paired *t*-test found a statistical difference for all variables in the two districts except for ‘referred’ in the non-pilot district before and after NHI MPs contracting. In comparing the two districts using the independent *t*-test assuming unequal variances, there were statistical differences in ‘referred’, ‘not referred’ and ‘follow-up’ except for the self-referral rate.

**TABLE 3:** Annual outpatient departments data elements and indicators for Tshwane National Health Insurance pilot district and Ekurhuleni district from June 2012 to May 2014.

Variable	Tshwane district			Ekurhuleni district			Tshwane versus Ekurhuleni†
	Pre-NHI	Post-NHI	Pre- versus post‡ NHI (*p*)	Pre-NHI	Post-NHI	Pre versus post‡ NHI (*p*)	*p*
Mean OPD headcount follow-up (follow-up)	37 393	57 788	< 0.0001*	10 640	27 251	0.0012*	< 0.0001*
Mean OPD headcount not referred new (self-referral)	6442	8644	< 0.0001*	8313	15 236	0.0012*	0.0001*
Mean OPD not referred new rate (self-referral rate)	52.7	48.8	0.0092*	61.4	52.9	0.0430*	0.3478
Mean OPD headcount referred new (referred)	8418	9636	< 0.0001*	8031	26 127	0.5362	0.0032*

OPD, outpatient departments; NHI, National Health Insurance.

* , statistically significant.

† , Independent *t*-test assuming unequal variances.

‡ , Paired *t*-test.

Table 4 shows a single ITSA comparison of pre- and post-NHI implementation in OPD headcounts for each district separately. There was an increase in base trend for ‘self-referral’ in both the pilot district (3387 [901, 5873] [*p* = 0.010]) and the non-pilot district (5399 [1889, 8909] [*p* = 0.004]) and ‘referred’ in the non-pilot district (21 010 [5407, 36 611] [*p* = 0.011]) but not in the pilot district. A change in base trend was also found in the non-pilot district for ‘referred’ (21 010 [5407, 36 611] [*p* = 0.001]) but not in the pilot district. However, for self-referral rate there was a decrease in both change in rate (−1.7 [− 2.1, −1.2] [*p* < 0.0001]) and change in trend (−1.5 [−1.8, −1.2] [*p* < 0.0001]) in the pilot district but not in the non-pilot district.

**TABLE 4:** Single interrupted time series analysis for Tshwane pilot and Ekurhuleni non-pilot districts.

Parameter	Tshwane NHI pilot project district						Ekurhuleni non-pilot project district					
	Coefficient	95% CI	Standard error	*p*	Durbin-Watson statistic		Coefficient	95% CI	Standard error	*p*	Durbin-Watson statistic	
					Original	Transformed					Original	Transformed
**OPD headcounts follow-up (follow-up)**					1.9	2.0					1.0	1.5
Base level (*β*_0_)	844	−2514, 4201	1609	0.6060	-	-	929	−1054, 2913	951	0.3400	-	-
Base trend (*β*_1_)	21 381	−8040, 50 804	14 105	0.1450	-	-	2910	−10 231, 16 052	6300	0.6490	-	-
Change in level (*β*_2_)	−1268	−4817, 2280	1701	0.4650	-	-	643	−2340, 3627	1431	0.6580	-	-
Change in trend (*β*_3_)	−423	−1500, 652	516	0.4202	-	-	1572	−9, 3153	758	0.0513	-	-
**OPD headcounts not referred new (self-referral)**					2.2	2.0					2.6	2.0
Base level (*β*_0_)	−155	−440, 130	137	0.2700	-	-		352	−13, 719	50	0.8950	-	-
Base trend (*β*_1_)	**3387**	**901, 5873**	**1191**	**0.0100**	**-**	**-**	**5399**	**1889, 8909**	**1682**	**0.0040**	**-**	**-**
Change in level (*β*_2_)	156	−146, 457	145	0.2950	-	-	−369	−785, 44	199	0.0770	-	-
Change in trend (*β*_3_)	0.5	−113, 116	55	0.9934	-	-	−16	−238, 204	106	0.8697	-	-
**OPD headcounts referred new (referred)**					0.9	1.9					2.0	1.9
Base level (*β*_0_)	67	−452, 585	249	0.7920	-	-	840	−964, 2645	866	0.3440	-	-
Base trend (*β*_1_)	−335	−3154, 2483	1351	0.8070	-	-	**21 010**	**5407, 36 611**	**7479**	**0.0110**	-	-
Change in level (*β*_2_)	151	−670, 973	394	0.7060	-	-	−1867	−3991, 258	1019	0.0820	-	-
Change in trend (*β*_3_)	217	−210, 645	205	0.3018	-	-	−1030	−2190, 131	555	0.0791	-	-
**OPD headcounts not referred new rate (self-referral rate)**					3.1	2.3					2.5	1.9
Base level (*β*_0_)	0.2	−0.2, 0.6	0.9	0.3390	-	-	1.4	−0.1, 2.8	0.7	0.0580	-	-
Base trend (*β*_1_)	3.8	−0.7, 8.4	2.2	0.0960	**-**	**-**	**−19**	**−30.0, −9.0**	**5**	**0.0010**	**-**	**-**
Change in level (*β*_2_)	**−1.7**	**−2.1, −1.2**	**0.2**	**< 0.0001**	-	-	−1.1	−2.6, 0.3	0.7	0.1210	-	-
Change in trend (*β*_3_)	**−1.5**	**−1.8, −1.2**	**0.1**	**< 0.0001**	-	-	0.2	−0.3, 0.8	0.2	0.3470	-	-

OPD, outpatient departments; NHI, National Health Insurance; CI, confidence interval

**FIGURE 1:**
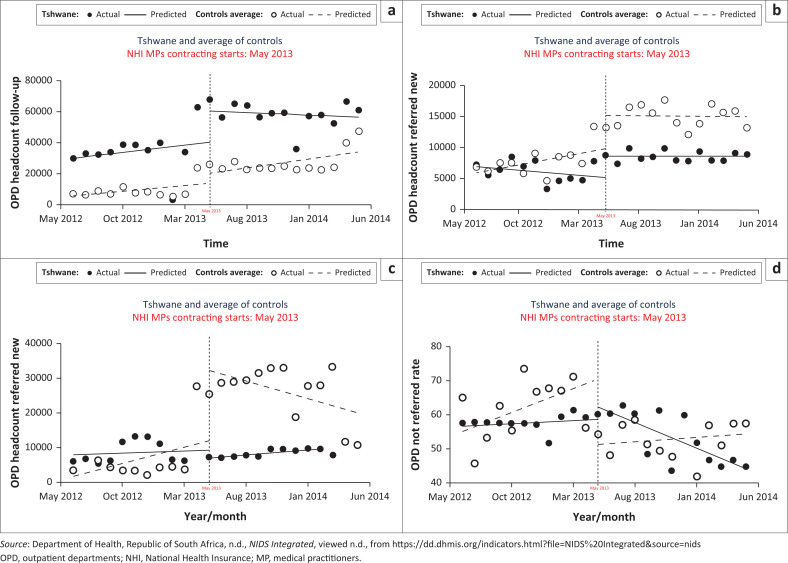
Controlled interrupted time series analysis graphs for outpatient departments headcounts. (a) OPD headcounts follow-up; (b) OPD headcounts new self-referral (c) OPD headcounts new referred (d) OPD headcounts new self-referral rate.

Table 5 and Figure 1 show differences in CITSA parameters of OPD headcounts between the pilot district (reference) and non-pilot district (control). There were differences in headcounts (24 382 [14 643, 34 121] [*p* < 0.0001]) for follow-up, trend (−516 [−969, −66] [*p* = 0.026]) and change in headcounts (529 [29, 1029] [*p* = 0.038]) for self-referral, change in trend (1293 [77, 2508] [*p* = 0.0376]) for referred and change in trend (−1.8 [−2.2, −1.1] [*p* < 0.0001]) for self-referral rate.

**TABLE 5:** Controlled interrupted time series analysis comparing Tshwane pilot and Ekurhuleni non-pilot districts

Parameter	Coefficient	95% CI	Standard error	*p*	Durbin-Watson statistic	
					Original	Transformed
**1. OPD headcounts follow-up:** Client attending a general or specialist outpatient clinic for follow-up care					1.7	1.9
Difference: Initial mean level (Base level) (β_4_)	**24 382**	**14 643, 34 121**	**4819**	**< 0.0001**	-	-
Difference: Mean baseline slope (Base trend) (β_5_)	223	−3511, 3956	1847	0.905	-	-
Difference: Pre-post trend (Change in level) (β_6_)	−1745	−5959, 2468	2085	0.407	-	-
Difference: Trend post-intervention (Change in trend) (β_7_)	−1520	−3200, 156	831	0.074	-	-
**2. OPD headcounts not referred new self-referral:** New clients attending a general or specialist outpatient clinic WITHOUT a referral letter from a PHC facility or a doctor					2.3	2.1
Difference: Initial mean level (Base level) (β_4_)	1008	−885, 2901	937	0.288	-	-
Difference: Mean baseline slope (Base trend) (β_5_)	**−516**	**−969, −66**	**223**	**0.026**	**-**	**-**
Difference: Pre-post trend (Change in level) (β_6_)	**529**	**29, 1029**	**247**	**0.038**	-	-
Difference: Trend post-intervention (Change in trend) (β_7_)	11	−233, 256	121	0.9247	-	-
**3. OPD headcounts referred new referred:** New client attending a general or specialist outpatient clinic with a referral letter from a PHC facility or a doctor					1.9	2.0
Difference: Initial mean level (Base level) (β_4_)	5915	−813, 12 643	3329	0.083	-	-
Difference: Mean baseline slope (Base trend) (β_5_)	−784	−2686, 1119	941	0.410	-	-
Difference: Pre-Post trend (Change in level) (β_6_)	**2077**	−187, 4340	1120	0.071	-	-
Difference: Trend post-intervention (Change in trend) (β_7_)	**1293**	**77, 2508**	**601**	**0.0376**	-	-
**4. OPD headcounts not referred new rate self-referral rate:** New OPD clients not referred as a proportion of OPD new clients.					2.7	1.9
Difference: Initial mean level (Base level) (β_4_)	1.5	−5.7, 8.8	3.7	0.685	-	-
Difference: Mean baseline slope (Base trend) (β_5_)	−1.2	−2.6, 0.1	0.7	0.076	**-**	**-**
Difference: Pre-post trend (Change in level) (β_6_)	−0.5	−1.9, 0.88	0.7	0.447	-	-
Difference: Trend post-intervention (Change in trend) (β_7_)	**−1.8**	**−2.2, −1.1**	**0.3**	**< 0.0001**	-	-

OPD, outpatient departments; PHC, primary healthcare; CI, confidence interval.

## Discussion

Our findings were based on ITSA, which compared pre- to post-intervention level and trends of OPD headcounts in a pilot and a non-pilot district separately and CITSA that compared OPD headcounts for follow-up, not referred new, not referred rate and referred new and trends between the two districts before and after NHI’s MPs contracting. Analysis of our findings using ITSA shows that after the NHI’s MPs contracting implementation, there was an increase in the trend for self-referral in both districts and for referred in the pilot district. However, there were decreases in both headcounts/rate and the trends in the pilot district and non-pilot district for self-referral rate. In comparing using CITSA, there were statistically significant differences in level of OPD headcounts follow-up and trends of OPD headcounts not referred new, referred new and self-referral rate between the two districts.

In comparing before and after MPs contracting using ITSA, there were statistically significant monthly increases in ‘self-referral’ of about 3400 clients in Tshwane district. Similarly, in Ekurhuleni district the monthly increase was about 5400. This confirms previous findings of bypassing of PHC services, potentially leading to overburdened district hospitals and underutilisation of PHC services because of self-referral, which can lead to wastage and poor allocation of resources (Masango-Makgobela et al. 2020:1–7). The differential findings between the two districts were supported by findings of monthly decreases for both self-referral rate and trend in the post-intervention period of 1.7 and 1.5 in the rate, respectively, in the pilot, but not in the non-pilot district. The findings were also confirmed by the CITSA findings of a difference in the monthly decrease in the post-intervention trend for the self-referral rate of 1.8 and a monthly increase of 1293 in the post-intervention trend for ‘referred’ in the pilot district compared with the non-pilot district. These CITSA findings of a reduction in self-referral and self-referral rate could be explained by the implementation of MPs contracting in the pilot district and are in line with findings in countries that have implemented some form of UHC with strong PHC, such as the United Kingdom, and contrast to those without, such as the United States of America (Forrest 2003:692–695). The mechanism of action could be improved perception of the quality of healthcare at the PHC level because of the presence of NHI contracted MPs, which has resulted in some clients not seeking primary care in hospitals as per the hypothesis of our study. Conversely, the increase in OPD headcounts referred new could be explained by MP’s referring clients to a higher level of care in response to increased workload at PHC level. There was an absence of an immediate increase but rather a gradual one post-intervention as evidenced by an absence of a level change in ‘self-referral rate’ but an increase in its post-intervention trend only (Groenewegen & Hutten 1991:1111–1119), or an identified need for a higher level care by MPs in clients historically treated by NPs.

The increase in the utilisation of OPD services post-MPs contracting is similar to those in countries that have implemented UHC programmes such as Brazil, Chile, Ghana, Mexico and Rwanda (International Alliance of Patient’s Organizations [IAPO] 2014). However, there are differences between the UHC models, epidemiological profiles, timing of evaluation and funding mechanism. For example, in Ghana, the National Health Insurance Scheme (NHIS) that began operations in 2005 was implemented by providing insurance for citizens to access care at public and private providers. Thus, in a study carried out in 2012 (7 years after initiation of NHIS), the UHC programme had led to increased utilisation of both outpatient and inpatient services. This study did not disaggregate PHC to OPD services even if both clinics and district hospitals were included in the study (Dalinjong et al. 2017:1–10). Similarly, in Rwanda, even if the Mutual Health Insurance (MHI) programme was scaled up in 2005, by 2007, an increase in the utilisation of health services was already noted, which coincided with a stabilisation and reduction in the HIV disease burden in the country (Kayirangwa et al. 2006:27–31).

The statistically significant CITSA difference in OPD follow-up headcounts between the two districts of more than 24 000 shows that despite having a lower population and higher medical insurance coverage, there is higher burden of disease on the district hospitals in the pilot district compared with the non-pilot district. This could be explained by a non-optimal down-referral mechanism from the district hospital to the PHC facilities in the pilot district (Hugo et al. 2020:e1–e10).

The findings of this study and those found in Singapore that also had private medical providers contracting as part of UHC interventions (Tan, Lee & Tan 2019:18–23) illustrate important lessons for the NHI in South Africa. There is a need to develop the capacity of PHC facilities and create organisational structures that would enhance coordination of activities between them and district hospitals by improving down-referral and supportive supervision of the PHC team by clinical specialists. This finding is crucial in addressing the immediate significant MP shortages and capacity at the PHC level and realising the broader vision of a single integrated system as envisaged by NHI.

There are several limitations to the interpretation of our findings. Firstly, the use of secondary data such as DHIS, which were not intended for the specified research question, may be incomplete and the interpretation limited. For example, we are not able to conclusively determine that the high number of follow-up headcounts in the pilot district is because of non-optimal down-referrals as there is no data collected for this indicator both at the PHC and OPD levels. However, the pre-selected data elements used in this study were complete in that they were reported for every month of the period under consideration. Secondly, the proximity of the two districts being compared could have led to population contamination, substitution and migration effects. However, this effect could also be considered negligible as the population growth in both districts was similar.

## Conclusion

Our findings suggest that the implementation of private MPs contracting as part of the NHI pilot project in PHC facilities in the Tshwane district may have resulted in the decrease in self-referrals at district hospitals. Thus, it could have resulted from an improvement in the perceived quality of healthcare at the PHC level. Despite a general reduction in the selected OPD headcounts, the follow-up at the district hospital level was high in the Tshwane district, suggesting an increased workload for staff at district hospitals after private MPs were contracted to work at PHC facilities.

## Recommendations

We recommend that with the implementation of NHI, the down-referral systems from district hospitals to PHC facilities and their capacity in managing conditions dictated by the epidemiological transition should be improved.
